# Altered Distribution of Circulating T Follicular Helper-Like Cell Subsets in Rheumatoid Arthritis Patients

**DOI:** 10.3389/fmed.2021.690100

**Published:** 2021-07-19

**Authors:** Rui Su, Yanyan Wang, Fangyuan Hu, Baochen Li, Qiaoling Guo, Xinyu Zheng, Yue Liu, Chong Gao, Xiaofeng Li, Caihong Wang

**Affiliations:** ^1^Department of Rheumatology, The Second Hospital of Shanxi Medical University, Taiyuan, China; ^2^Pathology, Joint Program in Transfusion Medicine, Brigham and Women's Hospital/Children's Hospital Boston, Harvard Medical School, Boston, MA, United States

**Keywords:** rheumatoid arthritis, follicular regulatory T cells, follicular helper T cells, B cells, subsets

## Abstract

**Objective:** Recent studies on follicular regulatory T (Tfr) and follicular helper T (Tfh) cells suggest that they may participate in the pathogenesis of rheumatoid arthritis (RA). Here, we examine Tfr-like and Tfh-like cells and their subsets in RA and assess the correlations between these subsets with B cells and cytokines related to the pathogenesis of RA and their clinical significance.

**Methods:** The study population consisted of 18 healthy controls and 47 RA patients (17 new onset, 57.00 ± 11.73 years; 30 treated RA patients, 57.56 ± 1.97 years). Disease activity scores in 28 joints were calculated. The positive rates of rheumatoid factor (RF) and anticyclic citrullinated peptide antibodies (anti-CCP) were 82.9 and 89.4%, respectively. Cell subsets were analyzed using flow cytometry, and serum cytokine levels were measured using cytometric bead array.

**Results:** Tfh-like and PD-1^+^ Tfh-like cells were elevated, and the distribution of Tfh-like cell subsets was altered with increased Tfh17-like and Tfh1/17-like cells in RA patients. The receiver operating characteristics curves for Tfh-like, Tfh17-like, Tfh1/17-like, and PD-1^+^ Tfh-like cells indicate improved RA diagnostic potential. RA patients had decreased regulatory T (Treg), Tfr-like, and memory Tfr-like (mTfr-like) cells and increased Tfh-like/Treg, Tfh-like/Tfr-like, and Tfh-like/mTfr-like cell ratios. Tfh-like cells and their subsets, including Tfh1-like, Tfh2-like, Tfh1/17-like, and PD-1^+^ Tfh-like cells, were positively correlated with B cells. Tfh-like/Treg, Tfh-like/Tfr-like, and Tfh-like/mTfr-like cell ratios were positively correlated with B cells in new-onset RA. Interleukin (IL)-2, IL-4, IL-17, interferon-γ, and tumor necrosis factor-α were positively correlated with Tfr-like and mTfr-like cells. IL-2 and IL-10 were positively correlated with Tfh-like and Tfh2-like cells. IL-4 was positively correlated with Tfh-like cells.

**Conclusions:** Tfh-like and PD-1^+^ Tfh-like cells are increased, whereas Treg, Tfr-like, and mTfr-like cells are decreased in RA, leading to an imbalance in Tfh-like/Treg, Tfh-like/Tfr-like, and Tfh-like/mTfr-like cell ratios. Tfh-like cells and a portion of their subsets as well as Tfh-like/Treg, Tfh-like/Tfr-like, and Tfh-like/mTfr-like cell ratios are closely related to B cells. Dysfunction of cell subsets leads to abnormal levels of cytokines involved in the pathogenesis of RA. The altered distributions of Tfh-like cell subsets, especially Tfh1/17-like cells, represent potential therapeutic targets for treatment of RA.

## Introduction

Rheumatoid arthritis (RA) is a chronic, systemic autoimmune disease of unknown etiology that is characterized by chronic inflammation and synovial infiltration of immune cells. Abnormalities of CD4^+^ T cells play major roles in the immunopathogenesis of RA (1, 2). The immune mechanism of RA has yet to be fully elucidated. Imbalance between T follicular helper cells (Tfh) and T follicular regulatory cells (Tfr)—two recently identified subsets of CD4^+^ T cells that participate in germinal centers (GCs) and regulate B cell proliferation and differentiation, thus playing important roles in maintaining immune homeostasis—may be important in the pathogenesis of RA (3–7). Tfr and Tfh cells are typically localized in follicles, and those in the blood are commonly referred to as Tfr-like and Tfh-like cells, respectively (8, 9).

A number of groups studying the functional properties of a human T cell subset marked by the expression of CXC chemokine receptor 5 (CXCR5) acting as a distinct memory T cell subset with B cell helper function designated these cells as Tfh cells (10, 11). Based on the differential expression of CXCR3 and CCR6, CD4^+^CXCR5^+^ T cells are classically divided into three major subsets: CXCR3^+^CCR6^−^ (Tfh1-like cells), CXCR3^−^CCR6^−^ (Tfh2-like cells), and CXCR3^−^CCR6^+^ (Tfh17-like cells) (5). However, many recent studies of Tfh-like cells in RA have focused on their relations to disease activity; typical antibodies, such as rheumatoid factor (RF) and anticyclic citrullinated peptide antibody (anti-CCP) (5, 12–17); and the effects of drug treatment on Tfh-like cell frequency (18–21). These studies suggest that Tfh-like cells are involved in the pathogenesis of RA and may be potential therapeutic targets. On the other hand, it has been suggested that these cells may be useful for predicting the response to disease-modifying antirheumatic drugs. However, the distribution of Tfh-like cell subsets and their relationships with B cells in RA remain unclear. In particular, there has been little research regarding the role of Tfh1/17-like cells in the pathogenesis of RA.

Treg cells are a subpopulation of regulatory T cells expressing the transcription factor FoxP3 that play a crucial role in the maintenance of immunological self-tolerance and homeostasis (22). Targeted Treg therapy has already been used to treat autoimmune diseases, but the characteristics and functions of different Treg subsets in autoimmune diseases have yet to be fully elucidated. Some groups have examined a subpopulation of follicular T cells designated as Tfr cells that can suppress the GC response, share phenotypic characteristics with Tfh and Treg cells but are distinct from both, and express both FoxP3 and Bcl6 (23, 24). CD45RA acts as a marker to distinguish native and memory T cells. CD45RA^−^ cells represent memory cells, whereas CD45RA^−^ memory Tfr-like (mTfr-like) cells are also considered activated Tfr-like cells. Gradually, it was realized that Tfr-like cells can interact with Tfh cells and B cells and inhibit the GC reaction to suppress production of high-affinity antibodies. Considering the relative difficulty in obtaining human samples of secondary lymphoid tissues, most studies focus on Tfr-like and Tfh-like cells in the peripheral blood. However, their characteristics and functions in peripheral blood are still not clear.

Age is an important risk factor for the development of RA, and accelerated immune aging has been demonstrated in RA (25). However, aging can lead to profound changes in the immune system, resulting in immune dysfunction. The correlations between frequencies of Tfr-like and Tfh-like cells and their subsets and age in RA have not been clarified.

The present study was performed to elucidate the roles of circulating Tfr-like and Tfh-like cells and their subsets in the pathogenesis of RA as well as their relations with B cells and cytokines associated with the pathogenesis of RA. We determined the percentages of Tfr-like, mTfr-like, Tfh-like, and Tfh-like cells and the Tfh1-like, Tfh2-like, Tfh17-like, and Tfh1/17-like cell subsets in the peripheral blood of RA patients and healthy controls using flow cytometry. We also analyzed the correlations between these subsets and B cells in new-onset RA patients. We further evaluated the correlations between age, the course of RA, indicators of disease activity [erythrocyte sedimentation rate (ESR), C-reactive protein (CRP), and disease activity score-28 considering the ESR (ESR-DAS28)], immunoglobulins (IgA, IgM, IgG), and typical antibodies of RA [RF, anti-CCP, anti-keratin antibody (AKA), and antiperinuclear factor (APF)] and these cell subsets. In addition, we analyzed the correlations between these cell subsets and serum levels of cytokines related to the pathogenesis of RA, including interleukin (IL)-2, IL-4, IL-6, IL-10, IL-17, interferon (IFN)-γ, and tumor necrosis factor (TNF)-α.

## Materials and Methods

### Subjects

The study population consisted of 47 patients diagnosed with RA (17 new-onset RA patients and 30 treated RA patients hospitalized due to disease relapse) according to the 1987 revised criteria of the American College of Rheumatology (26). All patients were hospitalized at the Department of Rheumatology from October 2018 to December 2019 in the Second Hospital of Shanxi Medical University. Of the 30 patients with RA who had been treated previously, eight were taking prednisolone (≤10 mg/per day) before blood samples were taken, four were taking disease-modifying antirheumatic drugs, and the remainder had stopped drug treatment for more than 3 months. Baseline characteristics and medications of the RA patients enrolled in the study are summarized in Supplementary Table 1. Patients with tumors or serious pathogen infections or those who were pregnant were excluded. We selected 18 healthy subjects as controls (healthy control group), who were age-matched with the RA group. Blood samples obtained from each of the subjects were used in the present study. T cell subsets were detected in 47 RA patients, and B cells were detected in 17 new-onset RA patients. Data on the clinical and serological parameters of these patients were collected, including routine blood test results, the ESR, and levels of CRP, IgG, IgA, IgM, RF, and anti-CCP antibody. Disease activity was assessed based on the ESR-DAS28. The study received approval from the Second Hospital of Shanxi Medical University ethics committee [approval no. (2019) YX No. (105)].

### Flow Cytometry for the Analysis of Lymphocyte Immunophenotypes

To determine the percentages of cell subsets, peripheral blood samples (6 mL) were collected from each subject in tubes.

### Detection of T Cell Subsets

**Figure 1:**
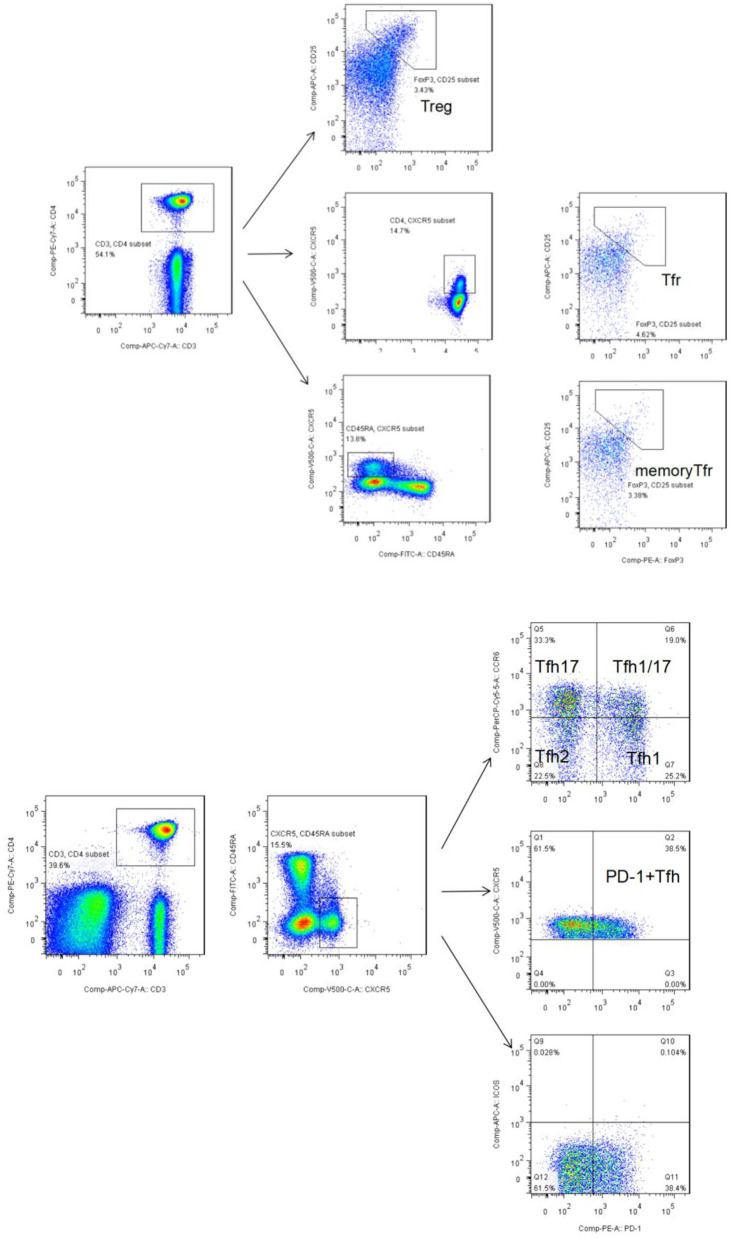
**(A)** Flow cytometry gating strategy for Tfr-like cells (CD3^+^CD4^+^ CD25^+^CXCR5^+^FoxP3^+^) and mTfr-like cells (CD3^+^CD4^+^CD25^+^CXCR5^+^CD45RA^−^FoxP3^+^). A total of 50,000 CD4^+^ T cells were collected. **(B)** Flow cytometry gating strategy for CD3^+^CD4^+^CXCR5^+^CD45RA^−^ Tfh-like cells, CD3^+^CD4^+^CXCR5^+^CD45RA^−^PD-1^+^ Tfh-like cells, and Tfh-like cell subsets, including Tfh1-like cells (CXCR3^+^CCR6^−^), Tfh2-like cells (CXCR3^−^CCR6^−^), Tfh17-like cells (CXCR3^−^CCR6^+^), and Tfh1/17-like cells (CXCR3^+^CCR6^+^). A total of 10,000 Tfh-like cells were collected. Tfr-like, T follicular regulatory-like; mTfr-like, memory T follicular regulatory-like; Tfh-like, T follicular helper-like; Treg, T regulatory.

Peripheral whole blood was aliquoted into two sample tubes, designated as tubes A and B. Tube A was used to detect Tfh-like cells. Briefly, the following were added to 200-μL aliquots of each blood sample followed by incubation at room temperature in the dark for 30 min: anti-CD3-APC-Cy7 (clone: SK7), anti-CD4-PE-Cy7 (clone: SK3), anti-CD45RA-FITC (clone: HI100), anti-CXCR5 (CD185)-BV510 (clone: RF8B2), anti-programmed cell death protein (PD)-1-PE (clone: EH12.1), anti-CXCR3-BV421 (clone: 1I6/CXCR3), anti-inducible T cell co-stimulator (ICOS)-AF674 (clone: DX29), and anti-CCR6-PreCP-Cy5.5 (clone: G034E3). Then, 2 mL of cell lysis solution was added, the sample solution was mixed and centrifuged, the supernatant was discarded, and the resultant pellet was washed with sheath liquid. Tfh-like cells (CD3^+^CD4^+^CXCR5^+^CD45RA^−^) and their subsets, including Tfh1-like cells (CXCR3^+^CCR6^−^), Tfh2-like cells (CXCR3^−^CCR6^−^), Tfh17-like cells (CXCR3^−^CCR6^+^), and Tfh1/17-like cells (CXCR3^+^CCR6^+^), were subjected to flow cytometry. A total of 10,000 Tfh-like cells per sample were collected (Figure 1A).

Tube B was used to detect Treg and Tfr-like cells. Briefly, the following were added to 200-μL aliquots of each blood sample followed by incubation at room temperature in the dark for 30 min: anti-CD3-APC-Cy7 (clone: SK7), anti-CD4- PE-Cy7 (clone: SK3), anti-CD45RA-FITC (clone: HI100), anti-CXCR5 (CD185)-BV510 (clone: RF8B2), and anti-CD25-APC (clone: 4E3). Then, 2 mL of cell lysis solution was added, mixed, centrifuged, the supernatant was discarded, and the resultant pellet was washed with buffer. Next, 250 μL of fixation/permeabilization concentrate and 750 μL of fixation/permeabilization diluent were added for intracellular staining according to the manufacturer's instructions (Invitrogen-Thermo Fisher Scientific). Anti-FoxP3-PE antibody (clone: 236A/E7) was added, and the mixture was incubated at room temperature for 30 min, followed by addition of 2 mL of 10× permeabilization buffer. A total of 50,000 CD4^+^ T events per sample were collected. Tfr-like cells were defined as CD3^+^CD4^+^CD25^+^CXCR5^+^FoxP3^+^ cells, and mTfr-like cells were defined as CD3^+^CD4^+^CD25^+^CXCR5^+^CD45RA^−^FoxP3^+^ cells (Figure 1B). Specific markers defining subpopulations and complete, detailed gating strategy in the study are summarized in Supplementary Table 2 and Supplementary Figure 1.

### Detection of B Cells

Blood samples were placed in tubes for immunofluorescence staining. To the tube was then added 20 μL of anti-CD3-FITC/CD19-APC antibody (BD Biosciences). The samples were incubated in the dark for 20 min at room temperature, washed with 1× FACS buffer, and then incubated for 15 min in the dark.

Fluorescence Minus One control and isotype-matched control antibodies were used in these procedures (Bio-Rad). All stained cells were analyzed using flow cytometry (FACSCanto II; BD Biosciences), and the data were analyzed using FACSDiva software and FlowJo V7.6.1 (Tree Star Inc., Ashland, OR, USA).

### Detection of Cytokine Levels via Cytometric Bead Array

IL-2, IL-4, IL-6, IL-10, IL-17, IFN-γ, and TNF-α were detected via cytometric bead array (Jiangsu Sage Biotechnology Co. Ltd.). Serum was isolated from peripheral blood and stored at −20°C. Cytokine standards were prepared using assay diluents via serial dilution. Capture beads were added to tubes containing samples, standards, and the negative control followed by incubation at room temperature in the dark for 2 h. The captured data of the standards and test specimens were transferred into BD FCAP Array software, and cytokines were assayed with the results expressed as pg/mL.

### Statistical Analysis

As the Shapiro–Wilk test revealed that the data had a nonnormal distribution, they are presented as the median and interquartile range (25 and 75th percentiles) and were compared using the Mann–Whitney U test. Correlation analysis was carried out using Spearman's rank test. Receiver operating characteristics (ROC) curves were plotted to explore the significance of Tfh-like cell subsets in RA. In all analyses, *p* < 0.05 was taken to indicate statistical significance. Statistical analyses were performed using SPSS 20.0 and GraphPad Prism version 8.0.

## Results

### Imbalance Among Tfh-Like Cells and Their Subsets in RA Patients

**Figure 2:**
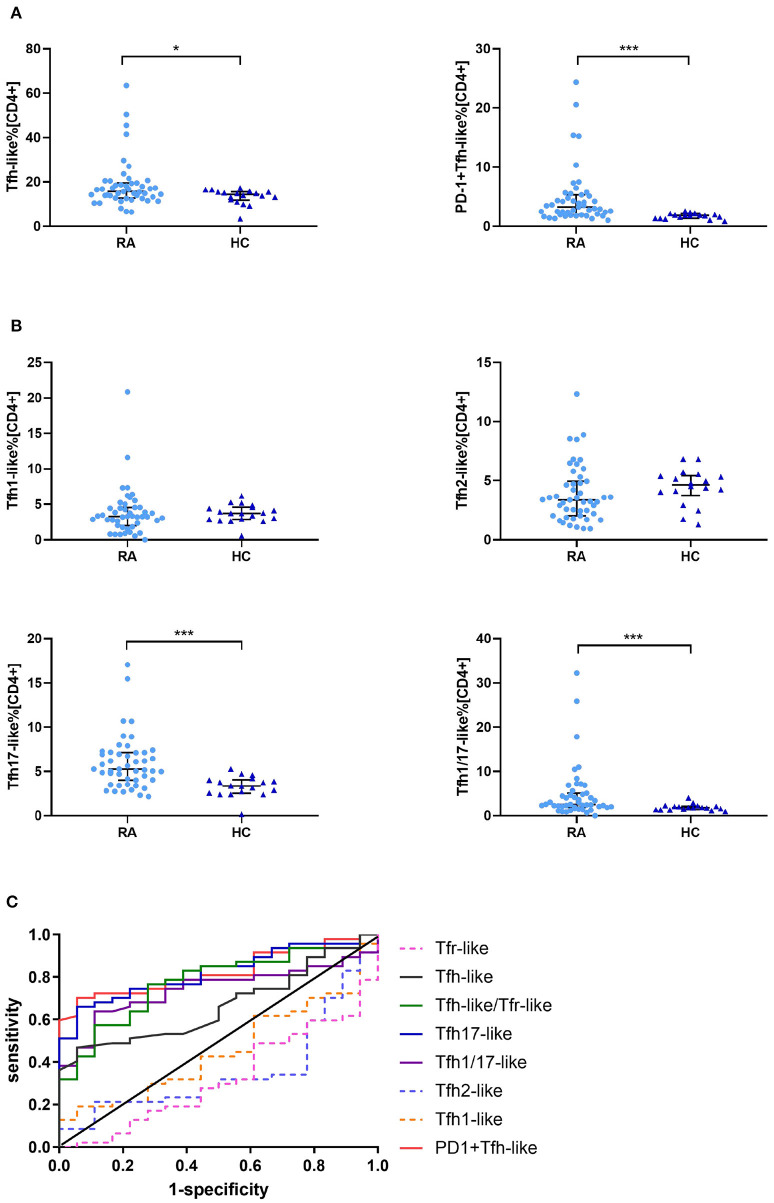
Tfr-like and Tfh-like cell subsets in RA patients and healthy controls. **(A)** Frequencies of Tfh-like and PD-1^+^ Tfh-like cells in patients with RA and healthy controls. **(B)** Frequencies of Tfh-like cell subsets in patients with RA and healthy controls. **(C)** The Tfh-like/Tfr-like cell ratio, Tfh-like cells, and Tfh17-like, Tfh1/17-like, and PD-1^+^ Tfh-like cell subsets as biomarkers of RA. Data in **(A,B)** are shown as the median and interquartile range. The Mann–Whitney *U*-test was used to compare the median values. *p < 0.05, ***p < 0.001. Tfr-like, T follicular regulatory-like; Tfh-like, T follicular helper-like; Treg, T regulatory.

We compared the percentages of peripheral blood CD33^+^CD4^+^CXCR5^+^CD45RA^−^ Tfh-like, PD-1^+^ Tfh-like, CXCR3^+^CCR6^−^ Tfh1-like, CXCR3^−^CCR6^−^ Tfh2-like, CXCR3^−^CCR6^+^ Tfh17-like, and CXCR3^+^CCR6^+^ Tfh1/17-like cell subsets (based on the expression of CXCR3 and CCR6) in healthy controls and RA patients. The results suggest that the levels of Tfh-like and PD-1^+^ Tfh-like cells were significantly increased in RA patients in comparison to healthy controls (Figure 2A). With regard to the distribution of Tfh-like cell subsets, RA patients exhibited significant elevation of Tfh17-like and Tfh1/17-like cell subsets compared with healthy controls (Figure 2B). To compare the significance of multiple Tfh-like cell subsets between RA and healthy controls in terms of identification of RA disease, we plotted ROC curves for these indicators (Figure 2C). The areas under the ROC curves (AUC) for Tfh-like, Tfh17-like, Tfh1/17-like, and PD-1^+^ Tfh-like cells were 0.667 [95% confidence interval (CI) 0.535–0.798], 0.823 (95% CI 0.723–0.922), 0.742 (95% CI 0.623–0.860), and 0.827 (95% CI 0.730–0.924), respectively. The sensitivities were 46.8, 66.0, 63.8, and 70.2%, respectively, and the specificities were 41.3, 94.4, 88.9, and 94.4%, respectively. These results suggest that imbalance in Tfh-like cells and their subsets may be involved in RA, and Tfh-like, Tfh17-like, Tfh1/17-like, and PD-1^+^ Tfh cells may be useful for monitoring the disease.

### Imbalanced Tfh-Like/Treg, Tfh-Like/Tfr-Like, and Tfh-Like/mTfr-Like Cell Ratios in RA Patients

**Figure 3:**
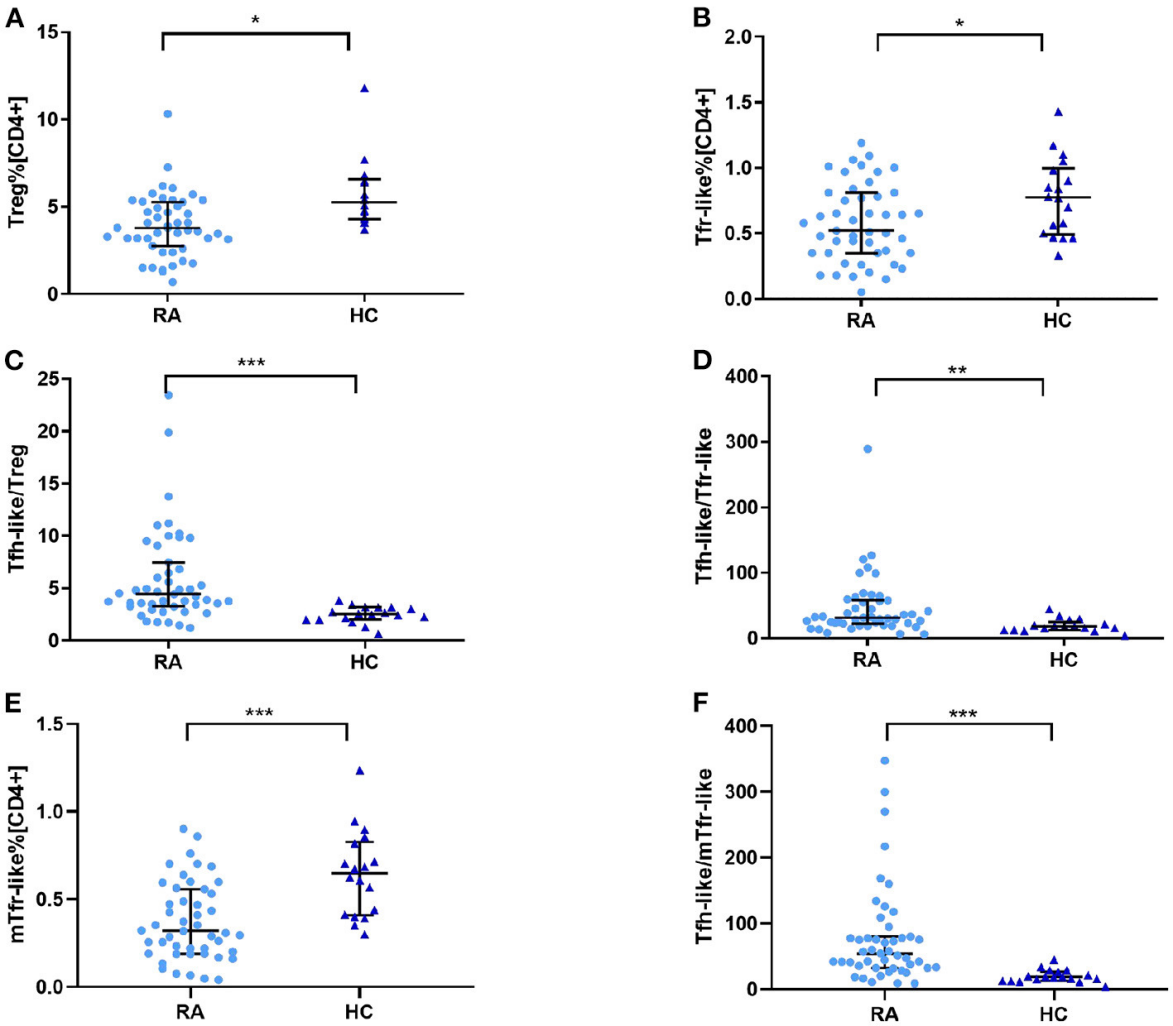
**(A)** Treg cell percentage [CD4^+^], **(B)** Tfr-like cell percentage [CD4^+^], **(E)** mTfr-like cell percentage [CD4^+^], and **(C)** Tfh-like/Treg, **(D)** Tfh-like/Tfr-like, and **(F)** Tfh-like/mTfr-like cell ratios in the peripheral blood of RA patients and healthy controls. Data are shown as the median and interquartile range. The Mann–Whitney U test was used to compare the median values. *p < 0.05, **p < 0.01, ***p < 0.001. Tfr-like, T follicular regulatory-like; mTfr-like, memory T follicular regulatory-like; Tfh-like, T follicular helper-like; Treg, T regulatory.

To determine whether Treg, Tfr-like, and mTfr-like cells also play important roles in the pathogenesis of RA, we examined the percentages of these cells in RA patients and healthy controls. The results indicate that RA patients had reduced numbers of Treg, Tfr-like, and mTfr-like cells in comparison to healthy controls (Figures 3A,B,E). The Tfh-like/Treg, Tfh-like/Tfr-like, and Tfh-like/mTfr-like cell ratios were all significantly elevated in RA patients in comparison with healthy controls (Figures 3C,D,F). In summary, the results suggest that imbalance in Tfh-like/Treg, Tfh-like/Tfr-like, and Tfh-like/mTfr-like cell ratios may play critical roles in the pathogenesis of RA.

### Tfh-Like Cells, Tfh1-Like, Tfh2-Like, Tfh1/17-Like, and PD-1^+^ Tfh-Like Cell Subsets, and Tfh-Like/Treg, Tfh-Like/Tfr-Like, Tfh-Like/mTfr-Like Cell Ratios Are Positively Correlated With B Cell Levels in New-Onset RA Patients

**Figure 4:**
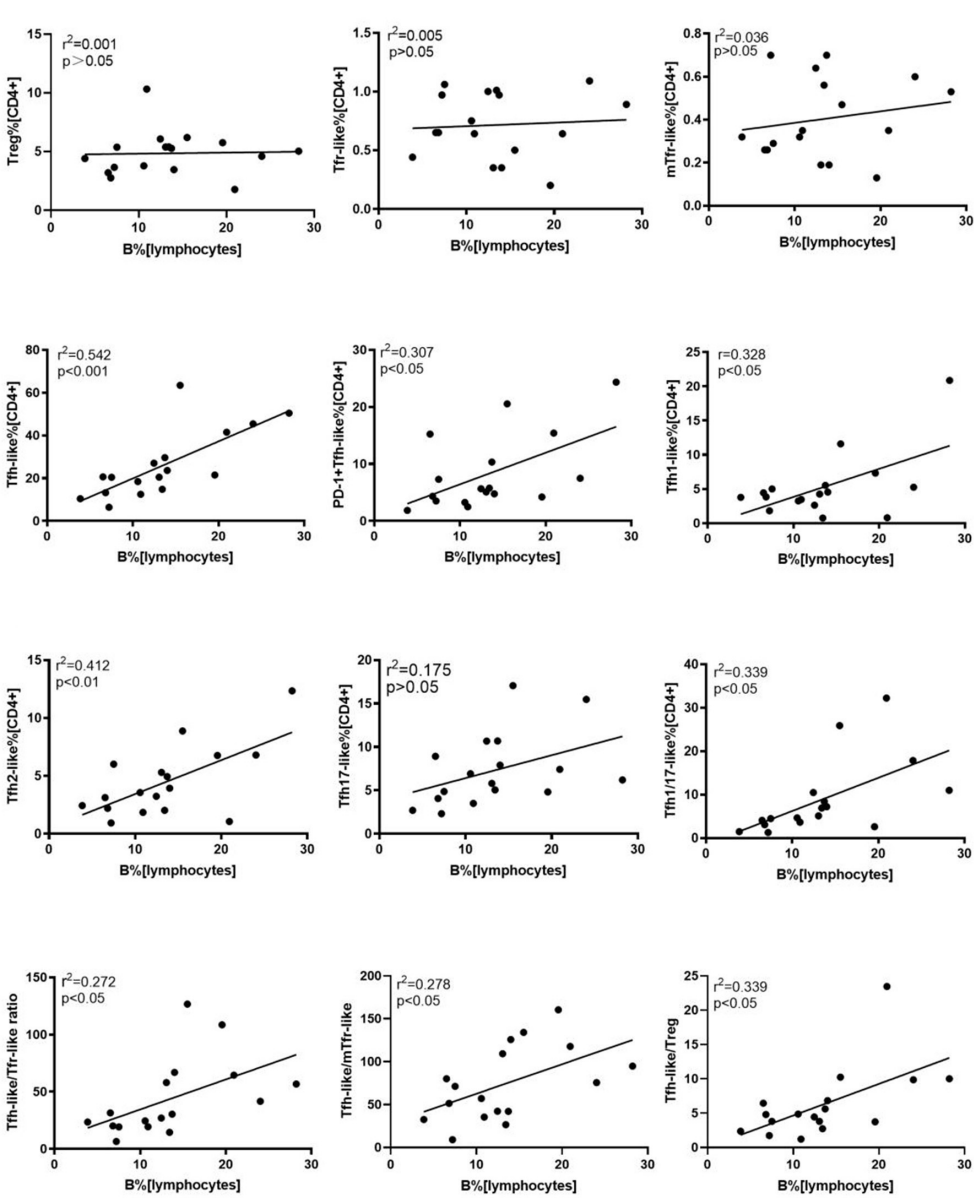
Correlation analysis of cell subsets, cell ratios, and B cells. Correlations were assessed using Spearman's rank test. *p* < 0.05 was taken to indicate statistical significance. A total of 10,000 Tfh-like cells were collected. Tfr-like, T follicular regulatory-like; mTfr-like, memory T follicular regulatory-like; Tfh-like, T follicular helper-like; Treg, T regulatory.

We further evaluated the correlations of Tfr-like and Tfh-like cells and their subsets with B cells in 17 patients with new-onset RA. The results indicate that Tfh-like cells and their subsets, including Tfh1-like, Tfh2-like, Tfh1/17-like, and PD-1^+^ Tfh-like cells, were positively correlated with B cells (Figure 4). There were no significant correlations between Treg or Tfr-like cells and B cells. However, the Tfh-like/Tfr-like, Tfh-like/mTfr-like, and Tfh-like/Treg cell ratios were all positively correlated with B cells in RA patients (Figure 4). No significant correlations were observed between Tfr-like, Tfh-like, and Tfh-like cell subsets and indicators of disease activity (ESR, CRP, DAS28), immunoglobulins (IgA, IgM, IgG), or typical antibodies of RA (anti-CCP, RF, AKA, APF) (Tables 1, 2). Only Tfh-like cells and the Tfh1-like and Tfh1/17-like cell subsets were positively correlated with antinuclear antibody (ANA) in RA patients (Tables 1, 2). In summary, imbalance in Tfh-like cells and a portion of their subsets as well as in Tfh-like/Treg, Tfh-like/Tfr-like, and Tfh-like/mTfr-like cell ratios was closely related to the level of B cells.

**Table 1:** Correlations of Tfr-like and Tfh-like cells with clinical and laboratory characteristics of RA patients.

	**Tfr-like**		**Tfh-like**	
	***r***	***p***	***r***	***p***
Age	0.157	0.290	0.346	0.017^*^
Course	0.007	0.963	0.179	0.228
IgA	−0.106	0.477	−0.122	0.448
IgG	−0.278	0.059	−0.116	0.472
IgM	0.031	0.764	−0.019	0.908
ESR	0.089	0.554	0.062	0.681
CRP	−0.003	0.985	−0.213	0.151
ESR-DAS28	0.152	0.308	0.093	0.534
RF	−0.123	0.410	−0.140	0.349
APF	0.199	0.179	−0.125	0.402
AKA	0.050	0.738	−0.199	0.180
Anti-MCV	0.150	0.316	−0.060	0.690
Anti-CCP	−0.007	0.962	−0.147	0.323
ANA	0.119	0.427	0.326	0.025^*^

*Correlations were assessed using Spearman's rank test*.

* *p < 0.05*.

*AKA, anti-keratin antibody; ANA, anti-nuclear antibody; anti-CCP, anti-cyclic citrullinated peptide; anti-MCV, anti-mutated citrullinated vimentin; APF, anti-perinuclear factor; CRP, C-reactive protein; DAS28, Disease Activity Score-28; RF, rheumatoid factor; ESR, erythrocyte sedimentation rate; Ig, immunoglobulin*.

**Table 2:** Correlations between Tfh-like cell subsets and the clinical and laboratory characteristics of RA patients.

	**Tfh1-like**		**Tfh2-like**		**Tfh17-like**		**Tfh1/17-like**	
	**r**	***p***	**r**	***p***	**r**	***p***	**r**	***p***
Age	0.028	0.852	0.228	0.124	0.423	0.003^*^	0.315	0.031^*^
Course	−0.147	0.325	0.038	0.799	0.250	0.090	0.105	0.484
IgA	−0.049	0.760	−0.121	0.450	0.027	0.867	0.132	0.411
IgG	−0.120	0.454	0.004	0.982	0.077	0.631	0.129	0.421
IgM	−0.060	0.711	0.113	0.482	−0.070	0.662	−0.063	0.695
ESR	−0.042	0.781	−0.045	0.765	0.177	0.235	0.285	0.052
CRP	−0.138	0.356	−0.060	0.691	−0.005	0.976	−0.084	0.573
ESR-DAS28	−0.214	0.148	−0.091	0.543	0.223	0.132	0.285	0.052
RF	−0.169	0.258	0.050	0.738	−0.023	0.880	−0.225	0.128
APF	−0.087	0.561	−0.043	0.775	−0.101	0.499	−0.019	0.901
AKA	−0.155	0.299	−0.131	0.380	−0.150	0.315	−0.121	0.417
Anti-MCV	−0.142	0.340	−0.101	0.499	−0.017	0.911	0.134	0.370
Anti-CCP	−0.109	0.466	−0.028	0.854	−0.068	0.650	−0.128	0.390
ANA	0.346	0.017^*^	0.185	0.212	0.075	0.614	0.363	0.012^*^

*Correlations were assessed using Spearman's rank test*.

* *p < 0.05*.

*AKA, anti-keratin antibody; ANA, anti-nuclear antibody; anti-CCP, anti-cyclic citrullinated peptide; anti-MCV, anti-mutated citrullinated vimentin; APF, anti-perinuclear factor; CRP, C-reactive protein; DAS28, Disease Activity Score-28; RF, rheumatoid factor; ESR, erythrocyte sedimentation rate; Ig, immunoglobulin*.

### Relations Between Age and T Cell Subsets

Aging can lead to profound changes in the immune system and result in immune dysfunction. We analyzed the correlations between age and the percentages of these different types of T cells in the blood. The results indicate that Tfh-like, Tfh17-like, and Tfh1/17-like cells (CXCR3^+^CCR6^+^) were positively correlated with the age of RA patients (Table 2). There were no significant correlations between other cells and age in RA patients. These results suggest that the frequencies of Tfh-like cell subsets are closely related to age in RA, and further studies of age-based stratification are, required.

### Relations Between Cytokine Levels and T Cell Subsets in RA Patients

**Figure 5:**
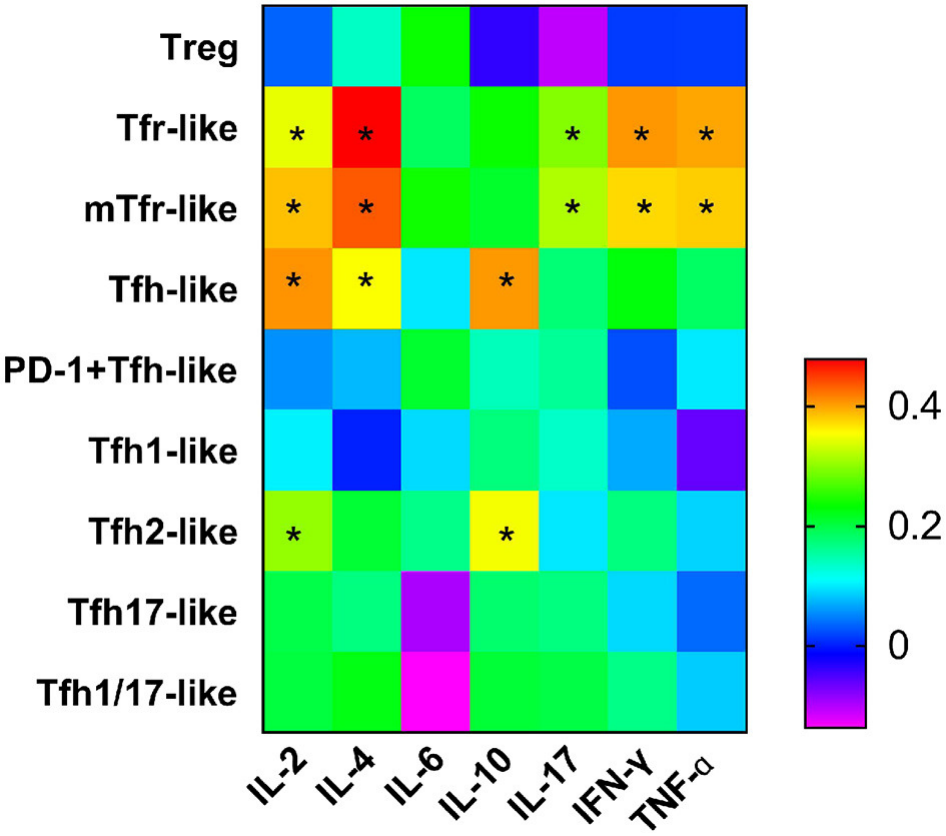
Heat map from correlation analysis between serum cytokines and Tfr-like and Tfh-like cells and their subsets. Correlations were assessed using Spearman's rank test. *p < 0.05. A total of 10,000 Tfh-like cells were collected. Tfr-like, T follicular regulatory-like; mTfr-like, memory T follicular regulatory-like; Tfh-like, T follicular helper-like; Treg, T regulatory.

We examined the correlations between levels of cytokines related to the pathogenesis of RA (IL-2, IL-4, IL-6, IL-10, IL-17, IFN-γ, TNF-α) and T cell subsets and found positive associations with Tfr-like and mTfr-like cells for IL-2 (*r* = 0.343, *p* = 0.018; *r* = 0.385, *p* = 0.007, respectively), IL-4 (*r* = 0.478, *p* = 0.001; *r* = 0.435, *p* = 0.002, respectively), IL-17 (*r* = 0.295, *p* = 0.044; *r* = 0.316, *p* = 0.013 respectively), IFN-γ (*r* = 0.405, *p* = 0.005; *r* = 0.373, *p* = 0.010, respectively), and TNF-α (*r* = 0.397, *p* = 0.006; *r* = 0.379, *p* = 0.009, respectively). In addition, positive correlations with Tfh-like and Tfh2-like cells were seen for IL-2 (*r* = 0.407, *p* = 0.005; *r* = 0.303, *p* = 0.038, respectively) and IL-10 (*r* = 0.404, *p* = 0.005; *r* = 0.350, *p* = 0.016, respectively), and IL-4 was positively correlated with Tfh-like cells (*r* = 0.353, *p* = 0.015). However, there were no significant correlations between the above cytokines and Treg cells (Figure 5). The frequencies of Tfr-like and Tfh-like cells were closely related to the levels of cytokines associated with the pathogenesis of RA. These findings suggest that dysfunction of cell subsets leading to abnormal levels of these cytokines may be involved in the pathogenesis of RA.

## Discussion

To examine the proportions of Tfr-like and Tfh-like cells and their subsets in RA, we compared the frequencies of these subsets between RA patients and healthy controls. The results reveal increases in the abundance of Tfh-like and PD-1^+^ Tfh-like cells and decreases in abundance of Treg, Tfr-like, and mTfr-like cells resulting in the imbalance of Tfh-like/Treg, Tfh-like/Tfr-like, and Tfh-like/mTfr-like cell ratios in RA. Further, alterations in the distribution of Tfh-like cell subsets were involved in the immunopathogenesis of RA, especially Tfh1/17-like cells, which were significantly elevated in RA patients compared with healthy controls and positively correlated with both the proportion of B cells and the level of ANA. In addition, correlations were also observed between Tfh-like cell subsets and serum cytokine levels.

Immune system disorders can influence the different stages of RA. In the inflammatory process of RA, immune homeostasis is often disturbed, leading to the generation of various abnormal autoantibodies, such as RF and anti-CCP. Tfr-like and Tfh-like cells can participate in GC formation and regulate B cell proliferation, differentiation, and antibody production (6), thus playing important roles in maintaining immune homeostasis. However, further exploration of Tfr-like and Tfh-like cells gradually yielded improved understanding of the mechanism of antibody production. Both Tfr-like and Tfh-like cells are suggested to be involved in the immunological pathogenesis of RA (3, 6). A number of groups report increased Tfh-like cells in RA (4, 12–15, 17), and specific staining also revealed Tfh-like cells among infiltrating immune cells in rheumatoid synovial tissues (27). However, there are only a few previous reports regarding the presence of Tfr-like cells in RA, and the results are controversial. For example, Liu et al. report that CXCR5^+^CD45RA^−^FOXP3^hi^ Tfr-like cells were increased in RA patients in stable remission in comparison to patients with active RA and healthy controls (4). Wang et al. also report increased CD4^+^CXCR5^+^CD127^lo^ Tfr-like cells among CD4^+^ T cells in RA patients compared with healthy controls (28). In the present study, we comprehensively evaluate the percentages of Tfh-like, PD-1^+^ Tfh-like, Tfr-like, mTfr-like, and Treg cells in RA patients and healthy controls. Our results reveal that Tfh-like and PD-1^+^ Tfh-like cells and Tfh-like/Tfr-like, Tfh-like/mTfr-like, and Tfh-like/Treg cell ratios were increased, whereas Tfr-like and mTfr-like cells were decreased in RA compared with healthy controls.

PD-1 and inducible T cell co-stimulators (ICOS) are commonly used as markers for identifying Tfh-like cells. In the present study, we stained CD3^+^CD4^+^CD45RA^−^CXCR5^+^ cells with antibodies against PD-1 and ICOS and found little or no ICOS expression. A previous study suggests that blood CXCR5^+^CD4^+^ T cells express few activation molecules, such as ICOS and CD69 (29). Tfh-like cells in the peripheral blood are not typical, and ICOS is seldom expressed in blood memory Tfh-like cells in contrast to the secondary lymphoid organs (30). Therefore, we hypothesize that ICOS expression may differ between Tfh-like and GC Tfh cells. Nevertheless, some studies suggest that ICOS are crucial for the differentiation of Tfh cells (31, 32). Of course, further *in vitro* and animal experiments are required to explore this issue. With progress in research on Tfh-like cells, Tfh-like cell subsets have attracted attention. To further determine the distributions of Tfh-like cell subsets, we evaluated the percentages of Tfh-like cell subsets in RA patients and healthy controls and found that Tfh17-like and Tfh1/17-like cells (CXCR3^+^CCR6^+^) were significantly elevated in RA patients compared with healthy controls. The expression of chemokine receptors is instrumental for defining human CD4^+^ T cell subsets. Blood CXCR5^+^CD4^+^ T cells consist of three subsets based on the expression of CXCR3 and CCR6, i.e., Tfh1-like (CXCR3^+^CCR6^−^), Tfh2-like (CXCR3^−^CCR6^−^), and Tfh17-like cells (CXCR3^−^CCR6^+^), but CXCR5^−^CD4^+^ T cells also consist of four subpopulations, including CXCR3^+^CCR6^+^ cells (29). Some groups report that the percentages of Tfh2-like and Tfh17-like cells in peripheral blood mononuclear cells were significantly elevated in RA patients with high disease activity compared with those with low disease activity (33). However, Costantino et al. report that there were no differences in Tfh1-like, Tfh2-like, and Tfh17-like cell subsets between RA and healthy controls (5). However, we stained CD3^+^CD4^+^CD45RA^−^CXCR5^+^ T cells with antibodies against CXCR3 and CCR6 and observed Tfh1/17-like cells (CXCR3^+^CCR6^+^) except Tfh1-like, Tfh2-like, and Tfh17-like cells. Tfh1/17-like cell populations have been reported in patients with tuberculosis (34), age-related macular degeneration (35), multiple sclerosis (36), and human immunodeficiency virus infection (37). These populations have also been observed in patients with IgG4-related disease and IgA vasculitis in children (38, 39), but there were no significant differences in levels compared with healthy controls. This subset may represent a group of poorly differentiated cells (31). CCR6^+^CXCR3^+^ “Th1/17” cells have been identified by the co-expression of the Th17 and Th1 lineage-specifying transcription factors RORγt and T-bet and share many features with Th1 and Th17 cells (40). Th1/17 cells can convert into CXCR3^+^CCR6^−^ and CXCR3^−^CCR6^−^ progeny *in vitro* (34). CCR6^+^CXCR3^+^ human memory cells are stable subsets, not transient phenotypes, that are actively selected for during memory T cell maturation *in vivo* (41). Interestingly, Th1/17 cells have distinct phenotypic and functional features, and are broadly reactive with various commonly encountered microorganisms (42). Furthermore, Th1/17 cells are thought to contribute to the pathogenesis of inflammatory bowel disease (43) and juvenile idiopathic arthritis (44). However, there have been few reports regarding Tfh1/17-like cells in RA. Our observations indicate that Tfh17-like and Tfh1/17-like cells were elevated in RA patients compared with healthy controls, but there were no differences in the percentages of Tfh1-like and Tfh2-like cells between the two groups. These observations suggest that the Tfh-like cell subset distribution is altered in RA, and that Tfh1/17-like cells may play an important role in the pathogenesis of this disease. Further exploration of the development and function of this population will help in determining how Tfh1/17-like cell responses are dysregulated during RA. In addition, ROC curve analysis of Tfh-like, Tfh17-like, Tfh1/17-like, and PD-1^+^ Tfh-like cells suggests that these subsets may be useful for monitoring the occurrence of the disease. Additional investigations are required to determine the precise roles of Tfh subsets in patients with RA.

Tfh and Tfr cells closely regulate B cell proliferation and differentiation and antibody production. Therefore, we further analyzed the correlations between these subsets and B cells. The results of the present study indicate that memory Tfh2-like and Tfh17-like cells in blood are able to induce naive B cells to produce immunoglobulins and switch isotypes through cytokine secretion, whereas blood memory Tfh1 cells lack this capacity (29, 45, 46). However, our results also indicate that Tfh-like cells and their subsets, including Tfh1-like, Tfh2-like, Tfh1/17-like, and PD-1^+^ Tfh-like cells, are positively correlated with B cells, but Tfh1-like and Tfh2-like cells were not significantly correlated with B cells, suggesting that the Tfh17-like and Tfh1/17-like cell subsets may play more critical roles in the interaction with B cells in RA. In addition, there was no significant correlation between Tfr-like cells and B cells, but the Tfh-like/Tfr-like, Tfh-like/mTfr-like, and Tfh-like/Treg cell ratios were all positively correlated with B cells. Therefore, the dysregulation of Tfh-like/Tfr-like, Tfh-like/mTfr-like, and Tfh-like/Treg cell ratios may be involved in the regulation of B cells, which may contribute to the pathogenesis of RA. Moreover, in the present study, disease activity indicators (ESR, CRP, DAS28), immunoglobulins (IgA, IgM, IgG), and typical antibodies of RA (anti-CCP, RF, AKA, APF) were not significantly correlated with Tfr-like, Tfh-like, or Tfh-like cell subsets. Only Tfh-like cells and their Tfh1-like and Tfh1/17-like cell subsets were positively correlated with ANA in RA patients. As all RA patients enrolled in our study were from the inpatient department, the average level of disease activity was high. Therefore, the effects of high disease activity level, sample size, and other factors cannot be excluded.

Immune function undergoes alterations with age, and the development and function of Tfr-like and Tfh-like cells change with age (47, 48). In the present study, it was interesting that Tfh-like, Tfh17-like, and Tfh1/17-like cells (CXCR3^+^CCR6^+^) were positively correlated with age in RA patients. These results suggest that the frequencies of Tfh-like cell subsets are closely linked to age in RA. The frequencies of blood Tfh-like cells are shown to be increased in the elderly population (49). Therefore, further studies to determine the pathogenic effects and mechanisms of age-related changes in Tfr-like and Tfh-like cells in RA as well as studies with age-based stratification are required.

Cytokines and immune cells are closely linked. However, the relations between the serum IL-2 levels and Tfr-like and Tfh-like cells have not been examined in detail. IL-2 is reported to inhibit the differentiation of Tfh cells via a mechanism that may involve the activation or inhibition of the STAT pathway and expression of Bcl-6/Blimp-1 (31, 50, 51). However, the effects of IL-2 on Tfr cells are not yet fully understood. Some groups suggest that IL-2 positively influences the differentiation of Tfr-like cells in the GC. However, some authors suggest that, unlike its effects on conventional Treg cells, IL-2 inhibits Tfr-like cell responses during influenza infection (52). In addition, our results show that the serum IL-2 level was positively associated with Tfr-like, mTfr-like, Tfh-like, and Tfh2-like cells. The positive correlation between IL-2 and Tfh-like cells appears to contradict the recently reported inhibitory effect of IL-2 on the differentiation of these cells. However, these results actually reconfirm the role of IL-2 as a pleiotropic cytokine. Maintaining the balance between the efficacy and pleiotropic functions of IL-2 is always a difficult problem in its use to treat disease (51). There may be some type of balance and feedback mechanism between IL-2 and Tfr-like and Tfh-like cells. The consumption of IL-2 by Tfr-like cells would lead to a low-IL-2 environment that is beneficial for the development of Tfh-like cells. Then, Tfh-like cells would increase along with Tfr-like cells, and the IL-2 level would also increase in a compensatory manner. The human immune system is extremely complex, and its components interact with one another to maintain immune homeostasis. It is difficult to determine their interactions in the complex background of the immune system, but it is clear that IL-2 is involved in the regulation of Tfr-like and Tfh-like cells. The relations between other cytokines and Tfr-like and Tfh-like cells were also evaluated. In addition, in this study, the anti-inflammatory cytokine IL-4 and the proinflammatory cytokines IL-17, IFN-γ, and TNF-α were positively correlated with Tfr-like and mTfr-like cells. IL-10 is a key cytokine involved in the suppression of immune responses although, in our study, the serum IL-10 level was positively related with Tfh-like and Tfh2-like cells. In fact, IL-10 has pleiotropic functions, and a recent study shows that IL-10 promotes the positive regulation of IgE responses to food antigens (53). It is clear that cytokines do not operate independently, but rather act in a network of activation and inhibition. Thus, the examination of individual cytokines often does not reflect the complexity of inflammation. In conclusion, the interactions between serum cytokines and these T cell subsets are involved in the pathogenesis of RA. Further studies to elucidate the regulatory mechanisms would be beneficial in the search for new therapeutic targets.

This study had some limitations, including the fact that all patients were drawn from a single medical center. In addition, due to the difficulty of *in vitro* separation culture, we did not conduct *in vitro* functional verification experiments. Furthermore, because it is challenging to obtain specimens and considering the ethical limitations of human trials, we did not evaluate the characteristics of Tfr-like and Tfh-like cells in the GC in RA patients.

The results of the present study suggest that the proportions of Tfh-like and PD-1^+^ Tfh-like cells are increased in RA, whereas those of Treg, Tfr-like, and mTfr-like cells are decreased, leading to imbalance in the Tfh-like/Treg, Tfh-like/Tfr-like, and Tfh-like/mTfr-like cell ratios. The altered distribution of Tfh-like cell subsets, especially Tfh1/17-like cells, suggests potential therapeutic targets for the treatment of RA. Dysregulation of the Tfh-like/Tfr-like, Tfh-like/mTfr-like, and Tfh-like/Treg cell ratios may be involved in the regulation of B cells. The dysfunction of cell subsets leads to abnormal levels of cytokines that are involved in the pathogenesis of RA. Additional studies of the changes in Tfh-like cell subsets and the relations with B cells and cytokines in RA may provide a better understanding of the pathogenesis of RA and facilitate the development of novel treatment strategies.

## Data Availability Statement

The Research Article data used to support the findings of this study are available from the corresponding author upon request.

## Ethics Statement

The studies involving human participants were reviewed and approved by Second Hospital of Shanxi Medical University. The patients/participants provided their written informed consent to participate in this study.

## Author Contributions

RS and YW performed all the experiments. RS performed the statistical analyses and prepared the manuscript. RS, YW, and FH participated in the acquisition of data. XZ, YL, BL, and QG contributed to sample collection. CG, XL, and CW participated in the design of the study. CW supervised the study and revised the manuscript. All authors were involved in drafting the article or revising it critically for important intellectual content, and all authors approved the final version to be published.

## Conflict of Interest

The authors declare that the research was conducted in the absence of any commercial or financial relationships that could be construed as a potential conflict of interest.
