# Supplementary material for: Altered Distribution of Circulating T Follicular Helper-Like Cell Subsets in Rheumatoid Arthritis Patients
Source: Front Med (Lausanne). 2021 Jul 19;8:690100. doi: 10.3389/fmed.2021.690100 (PMC8326448; doi:10.3389/fmed.2021.690100)
Supplement: Supplementary file 1 [file Table_1.docx]

**Supplementary Table 1.** Baseline characteristics of all RA patients and healthy controls

|  | Treated RA  (*n* = 30) | New-onset RA  (*n* = 17) | Healthy controls  (*n* = 18) |
| --- | --- | --- | --- |
| Sex (M/F) | 14/16 | 8/9 | 11/7 |
| Age (years) | 57.56 ± 1.97 | 57.00 ± 11.73 | 56.78 ± 12.89 |
| ESR (mm/h) | 60.64 ± 34.35 | 46.58 ± 32.48 | - |
| CRP (mg/L) | 34.27 ± 52.70 | 22.80 ± 33.63 | - |
| ESR-DAS28 | 7.75 ± 3.36 | 7.81 ± 3.64 | - |
| IgA (g/L) | 3.70 ± 1.35 | 3.35 ± 1.35 | - |
| IgG (g/L) | 14.31 ± 2.69 | 11.79 ± 2.75 | - |
| IgM (g/L) | 1.81 ± 1.11 | 1.51 ± 0.81 | - |
| WBC count (10^9^/L) | 7.01 ± 2.80 | 5.56 ± 1.70 | - |
| Hemoglobin (g/L) | 121.56 ± 21.40 | 123.16 ± 15.52 | - |
| Platelets (10^9^/L) | 296.80 ± 85.62 | 311.08 ± 109.33 | - |
| LY (10^9^/L) | 2.38 ± 3.03 | 1.25 ± 0.36 | - |
| ALT | 19.30 ± 16.94 | 20.17 ± 14.57 | - |
| AST | 21.43 ± 15.52 | 21.26 ± 9.52 | - |
| BUN (mmol/L) | 5.77 ± 2.54 | 4.84 ± 1.42 | - |
| Cr (μmol/L) | 60.74 ± 17.08 | 52.50 ± 12.66 | - |
| Pred (≤ 10 mg/per day) | 8/30 | - | - |
| DMARDs | 4/30 | - | - |

Clinical values represent the mean ± standard deviation.

Of 30 patients with RA who had been treated previously, eight were taking Pred (≤ 10 mg/per day) before blood samples were taken, four were taking DMARDs, and the rest had stopped drug treatment for more than 3 months.

ALT, alanine aminotransferase; AST, aspartate aminotransferase; BUN, blood urea nitrogen; Cr, creatine; DMARDs, disease-modifying antirheumatic drugs; LY, lymphocytes; Pred, prednisolone.
