# Supplementary material for: Altered Distribution of Circulating T Follicular Helper-Like Cell Subsets in Rheumatoid Arthritis Patients
Source: Front Med (Lausanne). 2021 Jul 19;8:690100. doi: 10.3389/fmed.2021.690100 (PMC8326448; doi:10.3389/fmed.2021.690100)
Supplement: Supplementary file 2 [file Table_2.docx]

**Supplementary Table 2.** Specific markers defining subpopulations

| Cell type | Markers |
| --- | --- |
| Tfr-like | CD3^+^CD4^+^CD25^+^CXCXR5^+^FoxP3^+^ |
| mTfr-like | CD3^+^CD4^+^CD25^+^CXCR5^+^CD45RA^−^FOXP3^+^ |
| Tfh-like | CD3^+^CD4^+^CXCR5^+^CD45RA^−^ |
| PD-1^+^ Tfh-like | CD3^+^CD4^+^CXCR5^+^CD45RA^−^PD-1^+^ |
| Tfh1-like | CD3^+^CD4^+^CXCR5^+^CD45RA^−^CXCR3^+^CCR6^−^ |
| Tfh2-like | CD3^+^CD4^+^CXCR5^+^CD45RA^−^CXCR3^−^CCR6^−^ |
| Tfh17-like | CD3^+^CD4^+^CXCR5^+^CD45RA^−^CXCR3^−^CCR6^+^ |
| Tfh1/17-like | CD3^+^CD4^+^CXCR5^+^CD45RA^−^CXCR3^+^CCR6^+^ |
| Treg | CD3^+^CD4^+^CD25^+^FoxP3^+^ |
| B | CD3^+^CD19^+^ |

A total of 10,000 Tfh-like were collected.

mTfr-like, memory T follicular regulatory-like; Tfh-like, T follicular helper-like; Tfr-like, T follicular regulatory-like; Treg, T regulatory.
