# Supplementary figures and images for: Altered Distribution of Circulating T Follicular Helper-Like Cell Subsets in Rheumatoid Arthritis Patients
Source: Front Med (Lausanne). 2021 Jul 19;8:690100. doi: 10.3389/fmed.2021.690100 (PMC8326448; doi:10.3389/fmed.2021.690100)

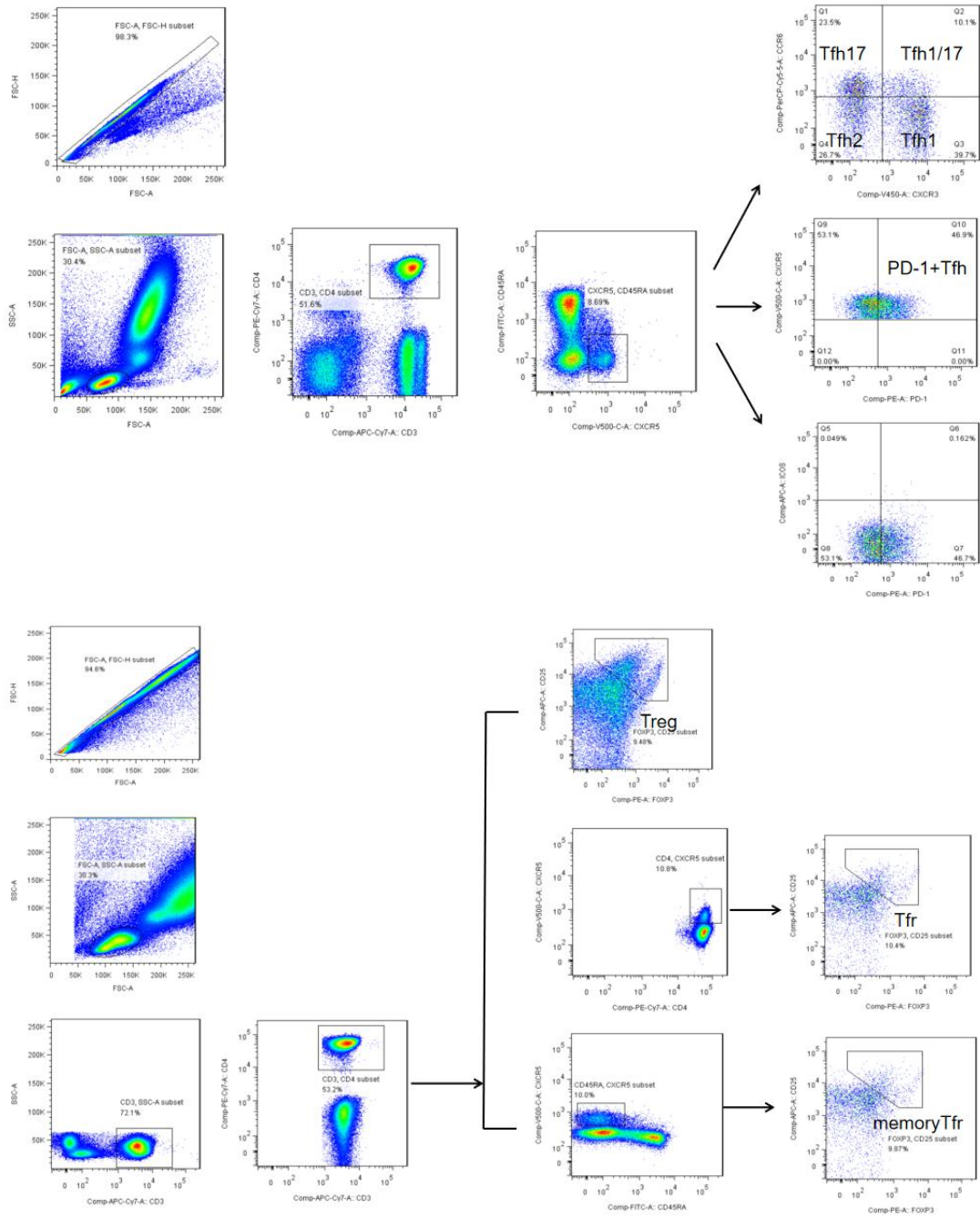

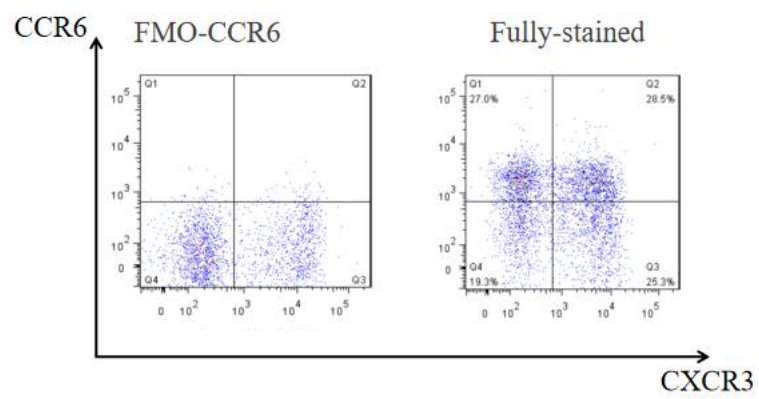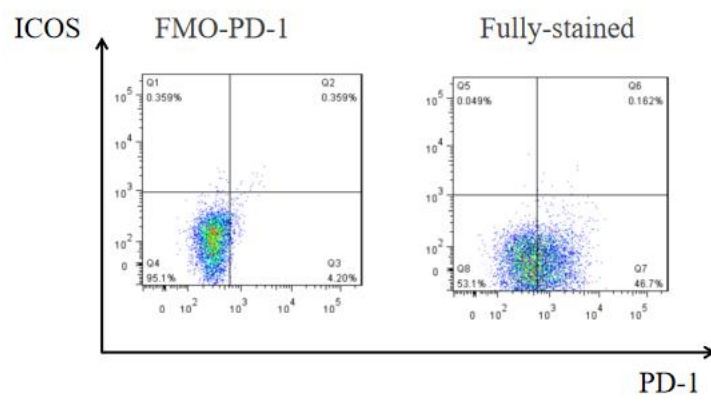

Supplementary figures. Complete, detailed gating strategy and the FMO of CCR6 and PD-1.

Supplement: Supplementary file 3 [file Image_1.pdf]
